# Occupational exposures, smoking and airway inflammation in refractory asthma

**DOI:** 10.1186/1471-2466-14-207

**Published:** 2014-12-19

**Authors:** Jodie L Simpson, Maya Guest, May M Boggess, Peter G Gibson

**Affiliations:** Centre for Asthma and Respiratory Disease, Faculty of Health and Medicine, The University of Newcastle, Callaghan, NSW Australia; Department of Respiratory and Sleep Medicine, Hunter Medical Research Institute, New Lambton Heights, NSW Australia; School of Health Sciences, Faculty of Health and Medicine, University of Newcastle, Callaghan, NSW Australia; School of Mathematical and Statistical Sciences, Arizona State University, Tempe, AZ USA

**Keywords:** Refractory asthma, Neutrophils, Occupational exposure

## Abstract

**Background:**

The influence of occupation and ex/passive smoking on inflammatory phenotype is not well understood. The aim of this study was to examine the relationship between occupation, past smoking and current passive smoking and airway inflammation in a population of adults with refractory asthma.

**Methods:**

Sixty-six participants with refractory asthma were characterised. Occupational exposure to asthma causing or worsening agents were identified with an asthma-specific job exposure matrix. Exposure to passive cigarette smoke was determined by questionnaire and exhaled carbon monoxide assessment. The carbon content of macrophages was assessed in a sub-group of participants.

**Results:**

Nineteen participants had smoked previously with low smoking pack years (median 1.7 years). Ex-smokers more commonly lived with a current smoker (26% vs. 9%, p = 0.11) and were more likely to allow smoking inside their home (26% vs. 4%, p = 0.02) compared to never smokers. Twenty participants had occupations with an identified exposure risk to an asthmagen; thirteen had exposures to irritants such as motor vehicle exhaust and environmental tobacco smoke. Sputum neutrophils were elevated in participants with asthma who had occupational exposures, particularly those who were diagnosed with asthma at a more than 30 years of age.

**Conclusions:**

Sputum neutrophils are elevated in refractory asthma with exposure to occupational asthmagens. In addition to older age, exposure to both environmental and occupational particulate matter may contribute to the presence of neutrophilic asthma. This may help explain asthma heterogeneity and geographical variations in airway inflammatory phenotypes in asthma.

## Background

Asthma is a common and chronic disorder of the airways induced by multiple stimuli including exposure to allergens, particulates and infectious agents. The inflammatory pattern observed in asthma is heterogeneous [1] and non-eosinophilic inflammatory patterns while common, are not responsive to inhaled corticosteroid therapy [2, 3]. The triggers of non-eosinophilic airway inflammation in asthma remain elusive and approximately 40% of adults with non-eosinophilic asthma have neutrophilic bronchitis with increased expression of neutrophil cytokines and proteases [4]. In community sampling, increased respiratory symptoms have been associated with occupational exposures [5] and workplace-exacerbated asthma is associated with a non-eosinophilic phenotype [6]. Knowledge is scant about the influence of occupational exposures on airway inflammation in patients with refractory asthma.

Work related asthma includes patients with sensitiser or irritant-induced asthma in the workplace (termed occupational asthma), as well as patients with pre-existing asthma worsened by work exposures (workplace-exacerbated asthma) [7]. In workplace-exacerbated asthma, patients have pre-existing or concurrent asthma that worsens by exposure to irritants, aeroallergens, changes in temperature or exercise [8–11]. Approximately 20% of working adults may have workplace-exacerbated asthma [12] and they experience more symptoms, require more medical care and have a reduced quality of life [13].

Recognising work related asthma is important in improving our understanding of the role of asthmagens in exacerbating symptoms of those already diagnosed with refractory asthma. Also in clinical trials designed to test the effectiveness of a new treatment modality, exposure to a workplace asthmagen may be a significant confounding factor for consideration.

The employment history of individuals often involves the changing of jobs or occupations, differing environments, varying levels of exposure and multiple sources of exposure over a lifetime. These present challenges to researchers engaging in exposure assessment and have limited the ability to establish firm cause and effect models [14]. Despite these challenges, many studies have established systematic approaches focusing on lifetime occupational exposures using Job Exposure Matrices [15, 16]. The most widely used job exposure matrix in asthma research is an asthma-specific job exposure matrix (AsthmaJEM) [17, 18].

In this study, we examined the relationship between occupational exposure to asthmagens, tobacco smoke exposure and airway inflammation in adults with refractory asthma. We tested the hypothesis that patients with asthma exposed to occupational asthmagens would be more likely to have neutrophilic bronchitis than those without exposure and that exposure to passive cigarette smoke would result in a worsening of neutrophilic bronchitis.

## Methods

### Study participants

Adults with refractory asthma [19] were recruited from the Ambulatory Care Service of the Department of Respiratory and Sleep Medicine at the John Hunter Hospital (New Lambton Heights, NSW, Australia) between 2004 and 2006. Participants comprised part of the screening population from a previous study [20]. Participants were excluded if they were currently smoking, had an exacerbation of their asthma or required antibiotics in the past four weeks. The Hunter Area Health Service and The University of Newcastle Human Research Ethics Committees approved this longitudinal study.

### Clinical assessments

Participants reported smoking history and passive smoking exposures. The asthma control score [21] and quality-of-life score [22] were assessed. Spirometry (KoKo PD Instrumentation, Louisville, CO, USA), combined bronchial provocation testing and sputum induction with hypertonic saline (4.5%) were performed. Sputum selected from saliva was dispersed using dithiothreitol, the suspension was filtered and a total cell count and viability of leucocytes ascertained [23]. Cytospins were prepared and stained with May-Grünwald Giemsa stain and a differential cell count was obtained from 400 non-squamous cells.

### Passive and active smoking exposures

Smoking status was assessed by questionnaire [24], exhaled carbon monoxide (eCO) and cotinine by reagent strip (NicAlert™, Nymox Pharmaceutical Corporation, St.-Laurent QC, Canada) [25]. All included participants had an eCO of less than 10 ppm confirming their current non-smoking status [26].

### Carbon content of macrophages

May-Grünwald Giemsa stained slides from 29 participants were screened under a 100X oil objective using an Olympus BX61 microscope. Photographs of 50 macrophages were then taken from each slide and used for analysis using ImageJ software [27]. Macrophages were not selected based on the presence of carbon particles, rather, once a macrophage was identified then a further 50 macrophages were assessed as they were identified in each field of view. Each macrophage image was cropped and converted into a black and white image with the carbon particles considered the darkest particles. The number, size and area of carbon particles were determined.

### Inflammatory phenotype

Participants were categoriesed according to granulocytic inflammatory phenotype as follows: eosinophilic (eosinophils >3%), neutrophilic (neutrophils >61%), paucigranulocytic (eosinophils <3% and neutrophils <61%) and mixed granulocytic (eosinophils >3% and neutrophils >61%) [1, 28].

### Occupational exposures

A full occupational history was recorded by questionnaire at interview, with information relating to position, industry and calendar year at the beginning and end of the occupation. All jobs with duration of at least three months were recorded. A six-digit code (Australian Standard Classification of Occupation) was assigned by an experienced coder using a coding program developed by the Australian Bureau of Statistics [29]. Jobs were subsequently translated into a four-digit code (International Standard Classification of Occupations 1988) utilising a concordance tool supplied by the Australian Bureau of Statistics. Jobs were linked to estimates of exposure to 22 agents using an asthma-specific job exposure matrix (AsthmaJEM) [17].

The AsthmaJEM was first merged on job codes to evaluate exposure (yes/no) to each of the 22 agents for each reported job. Exposures to 18 known asthmagens and four work environments with exposure to irritants or with low level exposure to chemicals or allergens were evaluated. Examples of the most frequent occupational asthmagens estimated by the AsthmaJEM are latex, bioaerosols, highly reactive chemicals, industrial cleaning agents, metal sensitisers, metal working fluids environments and textile production.

Job history information was used to create a dataset that assessed occupational exposure. Exposure was defined as the maximum exposure level of the study participant over their working life to one of five groups:

1. High-risk exposure to high molecular weight (HMW ≥1000 kD) agents (protein-derived agents)
2. High-risk exposure to low molecular weight (LMW ≤1000 kD) agents (reactive chemicals) antigens
3. High risk exposure to mixed environments or agents
4. Low risk possible exposure to other respiratory hazards
5. Not exposed (reference group)

### Statistical methods

Descriptive statistics are reported as counts and percentages for categorical variables and median and interquartile range (IQR) (25^th^ percentile-75^th^ percentile) for continuous variables. Fisher’s exact test was used to test for a univariate association of occupational exposure to any categorical variable. Wilcoxon rank-sum test was used to test for a univariate association of occupational exposure and Kruskal-Wallis test for diagnosis age and exposure to any continuous variable. Multivariable linear regression, with a random effect for participant, was used with the specific objective of detecting an association between exposure probability and clinical and inflammatory markers. Insignificant variables were removed from the full model to obtain the simplest model with greatest explanatory power. Significance was determined at the 5% level. All data manipulation and analysis were performed in Stata/MP Version 12 [30].

## Results

Sixty-six eligible participants participated in the study. Table 1 reports detailed demographic and clinical summary statistics. Participants were middle aged (median 60 years), atopic (76%) adults with moderate-severe airflow obstruction without well-controlled asthma (asthma control score >0.75) [31], despite being prescribed a high dose of inhaled corticosteroids (ICS) (median 2000 μg daily) consistent with a diagnosis of refractory asthma.

**Table 1:** **Descriptive statistics of demographic and clinical assessments at visit 1** (**sample size N** = **66**)

	Count/Median	Percentage/ IQR (25 ^th^ -75 ^th^ percentile)	Count N
Age (years)	59.9	49.2 – 65.7	66
Female	34	52%	66
Age of asthma diagnosis (years)	18.5	6.0 – 42.0	66
Currently Employed	43	65%	66
Ex-smoker	19	29%	66
Ex-smoker Pack years	1.7	0.50 – 5.00	19
Atopic asthma	50	76%	66
Past 12 months unscheduled doctors visit	38	58%	66
Past 12 months oral corticosteroids	28	42%	66
Past 12 months hospitalisation	5	8%	66
Asthma control score (ACQ)	1.41	1.00 – 2.14	66
Quality of Life Total Score	5.71	4.81 – 6.44	66
FEV_1_% predicted	69.4	53.0 – 82.4	66
FEV_1_/FVC	66.7	58.0 – 76.0	66
ICS dose/1000 (mg/day)^†^	2	1 – 2	65
PD_15_^‡^	4.84	1.39 – 14.26	42
Dose response slope^*^	2.59	0.76 – 5.59	53

Count and percentage displayed for categorical variables and median and IQR otherwise.

^†^Inhaled corticosteroids normalisation: 1 μg beclomethasone = 1 μg budesonide = 0.5 μg fluticasone.

^‡^PD_15_: provocation dose causing fall in FEV_1_ of ≥15% from baseline.

^*^Dose response slope: % fall FEV_1_/mL 4.5% saline.

### Active and passive smoking exposures

Nineteen (29%) participants had previously smoked, however with relatively low smoking pack years of 1.7 (IQR 0.5 - 5). Most participants allowed smoking outside of their homes and ex-smokers were significantly more likely to allow smoking inside their home compared to never smokers. Spending time in smoky places outside of the participants’ home was common and not different between ex-smokers and never smokers (Table 2).

**Table 2:** **Characteristics of active and passive smoking exposures**, **by smoking status**, **at first visit** (**N** = **66**)

	Never smoked		Ex-smoker		P
	N = 47		N = 19		
			Median	IQR	
Smoking (pack-years)			1.70	0.5 – 5.00	
**Passive smoking at home**					
Lives with one or more smokers	4	9%	5	26%	0.108
Smoking not allowed in home	12	23%	1	5%	0.156
Smoking allowed inside	2	4%	5	26%	0.018
Smoking allowed outside	34	72%	13	68%	0.770
**Passive smoking elsewhere**					
No time with smokers	18	38%	4	21%	0.252
Spends time indoors smokers	6	13%	3	16%	0.709
Spends time outdoors smokers	22	47%	12	63%	0.283
	**Median**	**IQR**	**Median**	**IQR**	
**Smoking biomarkers**					
eCO (ppm)	2	1 - 3	2	1 – 3	0.751
NicAlert™	1	1 - 1	1	0 – 2	0.441

### Occupational exposures

Table 3 shows the occupations of participants and potential exposures according to AsthmaJEM [17]. Of the 66 participants, 46 (69.7%) had no exposure to any identified asthmagen, holding occupations such as schoolteacher, office clerk or sales assistant. Of the remaining 20 participants, 11 had occupations with high-risk exposures to asthmagens, such as latex, cleaning products and wood dust, leaving nine participants with low risk exposures such as exhaust fumes. The single category with the highest number of participants was those exposed to motor exhaust fumes with nine (45%) of the 20 participants identified as having exposure to asthmagens.

**Table 3:** **Occupations of participants with exposures to asthmagenic agents according to AsthmaJEM** (**N** = **66**)

Level of risk	Agents	Total N	N	Occupations
**High risk**	**High molecular weight**			
	Animals	1	1	Dairy and livestock producers
	Latex	2	1	Nursing and midwifery professionals
			1	Institution-based personal care workers
	Bioaerosols	2	1	Dairy and livestock producers
			1	Machine-tool operators
	Total number with a high molecular weight exposure	4*		
	**Low molecular weight**			
	Highly reactive chemicals	3	1	Biologists, botanists, zoologists and related professionals
			1	Institution-based personal care workers
			1	Helpers/cleaners in offices, hotels etc.
	Industrial cleaning products	2	1	Institution-based personal care workers
			1	Helpers/cleaners in offices, hotels etc.
	Wood dusts	1	1	Carpenters and joiners
	Metal sensitizers	2	1	Tool-makers and related workers
			1	Machine-tool operators
	Total number with a low molecular weight exposure	6*		
	**Mixed environments or agents**			
	Metal working fluids	2	1	Tool-makers and related workers
			1	Machine-tool operators
	Agricultural	1	1	Dairy and livestock producers
	Textiles	1	1	Tailors, dressmakers and hatters
	High irritant peaks	2	2	Police officers
	Total number with exposure to mixed environments or agents	6*		
	**Total number with a high risk exposure**	11*		
**Low risk**	**Possible exposure to other respiratory hazards**			
	Irritants, but not high peaks	3	1	Carpenters and joiners
			1	Mining-plant operators
			1	Helpers/cleaners in offices, hotels etc.
	Motor vehicle exhaust fumes	9	2	Police officers
			1	Motor mechanics and fitters
			1	Railway brakers, signallers and shunters
			3	Car, taxi and van drivers
			1	Bus and tram drivers
			1	Lifting-truck operators
	Environmental tobacco smoke	1	1	Waiters, waitresses and bartenders
	Total number with exposure to other respiratory hazards	13*		
	**Total number with a low risk exposure**	**9**		
**No risk**	**Total number with no exposure to any respiratory hazard**	**46**		

^*^Totals do not add since some participants had multiple exposures and/or multiple occupations.

Some occupations encounter more than a single exposure and therefore participants may have exposures to more than one agent. In this study we found 4 participants with two exposures and a further 4 with three exposures. This means that the total in Table 3 is greater than the number of participants. The occupations with the highest number of exposures were cleaner, personal care worker, farmer and machine-tool operator.

Table 4 shows descriptive statistics of clinical outcomes and inflammatory markers by exposure and diagnosis age (30+ or <30 years of age at diagnosis). The dichotomised age of diagnosis was significantly related to exposure (Odds Ratio 3.4, P = 0.03), with an older age of diagnosis in the exposed group. Higher percentages of neutrophils are seen in the exposed groups, 27 versus 47% in the 30+ diagnosis age group and 42 versus 51% in the younger diagnosis age group, although the difference does not reach statistical significance with this small sample size. Participants diagnosed with asthma before the age of 30 had worse lung function (lower FEV_1_% predicted and FEV_1_/FVC), more airways hyperresponsiveness (lower PD_15_ and higher dose response slope) and were more likely to be atopic compared to those participants with refractory asthma who were diagnosed at more than 30 years of age (Table 4).

**Table 4:** **Descriptive statistics of clinical biomarkers**, **by occupational exposure and diagnosis age** (**count and percentage or median and IQR**)

	Diagnosis age 30+, unexposed		Diagnosis age 30+, exposed		Diagnosis age <30, unexposed		Diagnosis age <30, exposed	
***Observed first visit***	N = 14		N = 12		N = 32		N = 8	
Age	14	63 (60-69)	12	64 (62-69)	32	53 (44-63)	8	46 (37-52)
Female	14	9 (64%)	12	4 (33%)	32	19 (59%)	8	2 (25%)
Currently Employed	14	6 (43%)	12	6 (50%)	32	24 (75%)	8	7 (88%)
Years at work	14	30 (30-35)	12	30 (20-30)	32	25 (19-33)	8	25 (15-26)
Ex-smoker	14	5 (36%)	12	2 (17%)**	32	7 (22%)	8	5 (63%)**
Smoking at home	14	2 (14%)	12	2 (17%)	32	3 (9%)	8	0 (0%)
Smoky places	14	7 (50%)	11	8 (73%)	32	20 (63%)	8	8 (100%)** ^X^
Atopic	14	6 (43%)	12	9 (75%)	32	28 (88%)	8	7 (88%)^XX AA^
ICS dose/1000	14	2 (1.6-2.0)	12	2 (0.9-2.0)	31	2 (0.6-2.0)	8	2 (1.0-2.0)
%Macrophages w carbon inclusion	1	30	5	46 (46-48)	7	30 (20-52)	1	42
# Carbon inclusions/macrophage	1	2	5	4 (4.0-5.0)	7	3 (2-6)	1	4
Total number Carbon inclusions	1	57	5	184 (160-212)	7	107 (27-224)	1	186
***Obs. multiple visits***	**N = 22**		**N = 20**		**N = 71**		**N = 14**	
FEV_1_% predicted	17	82 (59-88)	16	75 (64-84)	46	63 (48-79)	10	74 (67-83)^X AA^
FEV_1_/FVC	17	76 (64-80)	16	71 (67-77)	46	64 (56-69)	10	69 (63-71)^XX AA^
PD_15_	10	15 (12-29)	5	21 (19-34)	32	5 (1.1-8.6)	8	2 (1.3-6.5)^XX AA^
Dose response slope	14	1 (0.5-2.2)	11	0 (0.2-0.8)	41	3 (1.2-11.5)	9	6 (2.1-8.6)^XX AA^
Total cell # ×10^6^/mL	21	3 (2.1-3.6)	20	6 (2.7-10.6)	69	3 (2.1-7.8)	14	3 (1.8-7.2)
Macrophages, %	22	51 (28-72)	20	28 (15-51)	71	44 (20-61)	14	44 (28-52)
Neutrophils, %	22	27 (14-40)	20	47 (37-74)	71	42 (27-72)	14	51 (36-67)^XX^
Lymphocytes, %	22	1 (0.0-2.0)	20	0 (0.0-1.1)	71	1 (0.3-2.0)	14	0 (0.0-1.3)
Eosinophils, %	22	1 (0.3-3.3)	20	2 (0.9-9.9)	71	1 (0.3-2.8)	14	1 (0.3-1.8)
Col. epithelial cells, %	22	6 (1.5-12.8)	20	3 (0.8-7.0)	71	3 (1.5-5.9)	14	4 (2.5-7.3)
Squamous cells, %	22	5 (1.5-11.3)	20	2 (0.9-4.4)	71	3 (1.0-6.1)	14	5 (1.2-9.5)

Test of effect of exposure in each diagnosis age group: **significant P < 0.05. Fisher’s exact and rank-sum tests used.

Test of effect of exposure and diagnosis age group: ^X^marginally significant P < 0.10, ^XX^significant P < 0.05. Fisher’s exact and Kruskal-Wallis tests used.

Test of effect of diagnosis age group only: ^AA^significant P < 0.05. Fisher’s exact and rank-sum tests used.

Multivariable linear regression models were fit to assess the effect of possible confounders, such as age, gender and smoking characteristics, on the relationships between exposures and sputum inflammatory cells. More precisely, sputum neutrophils, eosinophils, macrophages and lymphocytes were examined and for each of these a model was fit including one of four exposure variables (high weight, low weight, mixed and all), the possible confounders and their interactions as explanatory variables. The aim of was to find any significant association between an exposure variable and a single inflammatory cell type. Of the many models fitted, only exposure, diagnosis age and age had a significant effect on sputum neutrophils. Of the different types of exposures, only combined exposure, that is, exposure to any agent, reached statistical significance in any model. Consequently, in the results detailed below, “exposure” means “exposure to any agent”.

The percentage of neutrophils was significantly affected by exposure, as were age (P = 0.001) and diagnosis age (a square root transform applied to the response to improve the normality of the residuals). In this model no smoking characteristics were significant (all P > 0.1) and neither was gender (P = 0.5). There were no significant interactions between exposure, diagnosis-age and age (P > 0.3). There was a significant interaction between age and the dose of inhaled corticosteroid (P = 0.007), for which the coefficient was negative, indicating that the effect of inhaled corticosteroids on neutrophil proportion is less at older ages. Being diagnosed with asthma over the age of 30 and having an occupational exposure was associated with a 20% increase in neutrophils compared to those without an occupational exposure.

**Figure 1:**
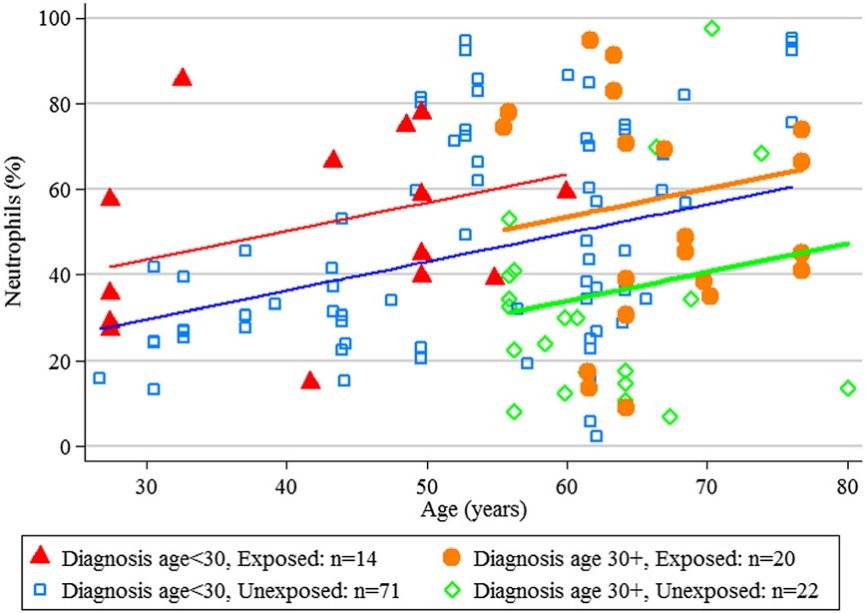
**Observed neutrophils (%) and estimated mean neutrophils (%) at corticosteroid dose 2000 μg, by age, diagnosis age group and occupational exposure (126 observations on 65 participants).**

Figure 1 shows the estimated average neutrophil percentage by age for exposure and diagnosis age groups. The difference between the exposed and unexposed in the diagnosis age group 30+ was significant (P = 0.032), but not the difference between exposed and unexposed in the younger diagnosis age group (P = 0.13). The average increase in neutrophil percentage associated to exposure is 20% in those diagnosed after the age of 30. The average increase in neutrophil percentage associated to exposure is 10% in those diagnosed under the age of 30 years. Neutrophil proportion increases with age at the rate of 0.5% per year of age. These results are displayed in Figure 1 for a dose of 2000 μg beclomethasone equivalents (see Figure 1).

## Discussion

This study examined the relationship between occupational exposures to asthmagens, age at diagnosis and airway inflammation in a population of adults with refractory asthma. We sought to test the hypothesis that patients with refractory asthma exposed to occupational asthmagens would be more likely to have neutrophilic bronchitis than those without exposure. We found that approximately one third of adults with poorly controlled asthma had occupations with identifiable exposures to occupational asthmagens whose symptoms may exacerbate or worsen their asthma. A diagnosis of asthma at more than 30 years of age was associated with a 20% increase in sputum neutrophils and 70% of participants in the exposed group showed evidence of airway inflammation, half of those with neutrophilic bronchitis.

The findings of the European Community Respiratory Health Surveys I and II investigated the association between occupational exposure and adult-onset asthma and asthma control. Survey I found that uncontrolled adult onset asthma was positively associated to exposure to an occupational asthmagen (and more so if the exposure was long term) and that the association was predominantly explained by the exacerbation domain suggesting those with exposure to occupational asthmagens experience more asthma exacerbations [15] Survey II, which investigated the association between 12 month and 10 year occupational exposures and adult-onset asthma have been published finding that the association was stronger for long-term exposures [15].

The role of neutrophils in asthma is controversial. We and others have reported the presence of neutrophilic asthma subtypes in adults with these participants being significantly older than those with normal proportions of neutrophils [1]. In adults, sputum neutrophils are associated with age and a neutrophilic phenotype of asthma is evident in older age even after correcting for the age related increase in neutrophils [32], suggesting there is something in addition to the effect of ageing that elevates sputum neutrophils in asthma. Smoking is an obvious consideration as it is known to induce a neutrophilic inflammation that can persist despite cessation, however in this study participants had smoked very little and current smoking or excessive past smoking is unlikely to have influenced sputum neutrophilic inflammation.

The influence of passive smoking on airway inflammation in asthma is less clear, especially in adults. In this study we observed that participants were generally tolerant of others smoking outside their homes and many spent time outdoors with smokers indicating the potential for significant passive smoking exposure. Ex-smokers were more likely to allow smoking inside their homes and often lived with other smokers, so despite not actively smoking these participants may have exposure to more environmental tobacco smoke. Exposure to environmental tobacco smoke has been associated with increased risk of COPD in those who have never actively smoked cigarettes [33], suggesting that exposure to passive smoke can influence airway inflammation and further studies are needed to understand the long term effects of exposure to environmental tobacco smoke exposure in adults with asthma.

Motor exhaust fumes were the most common exposure identified from the participants’ occupation analysis. This may represent a common exposure in adults with neutrophilic bronchitis as exposure to diesel exhaust can result in a neutrophilic infiltrate even in healthy individuals [34]. Exposure to motor exhaust fumes that result from living in close proximity to a highway or major road not only increases asthma risk but is also associated with neutrophilic bronchitis. Indeed the study of Wallace *et al* demonstrated that those living within 1 km of a major road were 4.7 times more likely to have neutrophilic bronchitis [35]. Similarly workplace-exacerbated asthma is more commonly associated with engine exhaust fumes than those with occupational asthma [6, 36]. While neutrophilic asthma is common [37–39], in some centres very few patients exhibit neutrophilic bronchitis [40]. Rossall *et al* compared the neutrophil counts in early- and late-onset asthma patients, finding that raised sputum neutrophil counts were present in those study participants with late-onset asthma, however not in healthy controls [41]. The authors went on to speculate that, as shown in earlier studies, other factors such as environmental pollution or infection were important in driving the neutrophilic airway inflammation observed in late-onset asthma [42, 43]. It is therefore possible that the common exposure may be motor exhaust fumes from either residential exposure and/or occupational exposure and further work is needed to examine this hypothesis.

### Implications for clinical practice

Recently, numerous researchers [44, 45] have suggested that asthma relating to occupation often goes unrecognised in clinical practice. While our research findings are not able to attribute asthma causation to the occupational exposures reported, they do however highlight the importance of taking into account that occupational exposures can exacerbate existing asthma. In addition our findings reinforce Cullinen and Cannon’s suggestion that “it is good practice to enquire into the employment of every working-age adult with asthma or rhinitis, particularly in those presenting with new symptoms or symptoms that have become more difficult to manage”. Patients should routinely be asked whether their symptoms improve when they are not at work” [45]. We would suggest in addition that it would be prudent to determine if the patient’s work includes exposure to known asthmagens.

### Implications for asthma patients

While some have postulated that occupational risk factors should be quickly identified to prevent uncontrolled asthma others suggest that, at least for younger adults with asthma, career choice should be an informed decision that takes into account their risks relating to asthma control. Our finding that 30% of participants in this study with refractory asthma had an occupation with an exposure known to either be associated with asthma risk or known to exacerbate existing asthma reinforces the concept of disease burden relating to occupational exposures.

On the other hand, we found that the majority of study participants had no identifiable risk and this may represent the healthy worker effect, where the presence of asthma has influenced job selection away from high-risk jobs and that our findings may underestimate the level of risk [44]. This is indeed an important point to consider, especially in light of the recent findings of Bhinder et al [46]. In a population of young Canadian adults with asthma, knowledge relating to the occupational risks for asthma and high-risk occupations was assessed, as well as their perception of the role of asthma in career choice. They found that young adults with asthma have suboptimal awareness of potential work-related asthma risks. With their family physician being most commonly the provider of their asthma care, few young adults reported talking to their family physician about the risks career choices could have on their asthma. This observation represents an area of asthma care that needs to be explored in young adults with asthma.

### Implications for researchers

The findings of this study highlight the importance of assessing occupational exposures of patients participating in clinical trials because the effectiveness of any new treatment modality may be underestimated if the role of an occupational asthmagen goes unrecognised [47]. In addition our study supports the recommendation by Papadopoulos [47] that detailed phenotyping/endotyping stands out as widely required in order to arrange or re-categorize clinical syndromes into more coherent, uniform and treatment-responsive groups.

### Study strengths and limitations

The strength of this study was the carefully characterised asthma and analysis of sputum samples, eCO and passive smoking exposures for the 66 participants. A limitation was the use of AsthmaJEM, which did not include a breakdown of exhaust fumes into diesel and gasoline but rather grouped all forms of exhaust singularly as exhaust fumes. A further limitation is that all participants were taking high doses of inhaled corticosteroids and further studies are needed to determine the effect of occupational exposures in participants with milder disease who do not require treatment with inhaled corticosteroids. Inhaled corticosteroids are known to enhance the survival of airway neutrophils [48] and increase following introduction of inhaled cortiocsteroids [40].

## Conclusion

Sputum neutrophils are elevated in refractory asthma with exposure to occupational asthmagens. In addition to older age, exposure to both environmental and occupational particulate matter may contribute to the presence of neutrophilic asthma. This may help explain asthma heterogeneity and geographical variations in airway inflammatory phenotypes in asthma.

## Abbreviations

AsthmaJEM: Asthma-specific job exposure matrix
eCO: exhaled carbon monoxide
HMW: High molecular weight
LMW: Low molecular weight
IQR: Interquartile range
ICS: Inhaled corticosteroid
FEV_1_: Forced expiratory volume in 1 second
FVC: Forced vital capacity
PD_15_: Provocation dose causing a 15% fall in FEV_1_
COPD: Chronic obstructive pulmonary disease
NHMRC: National health and medical research council
ACQ: Asthma control questionnaire
ppm: parts per million.
